# Factors Influencing Purchase Intention for Low-Sodium and Low-Sugar Products

**DOI:** 10.3390/foods9030351

**Published:** 2020-03-18

**Authors:** Yeowoon Park, Dongmin Lee, Seoyoung Park, Junghoon Moon

**Affiliations:** 1 Department of Agricultural Economics & Rural Development, Seoul National University, Seoul 08826, Korea; qkrdudns1101@snu.ac.kr (Y.P.); syp1130@gmail.com (S.P.); 2 Department of Food Processing and Distribution, Gangneung-Wonju National University, Gangneung 25457, Korea; dongminlee@gwnu.ac.kr

**Keywords:** cognitive dissonance theory, unhealthy = tasty intuition, food neophobia, low-sodium, low-sugar

## Abstract

As sodium and sugar intake in South Korea exceed recommended levels, the government and food industry have been attempting to reduce the amount of sodium and sugar in the food products. In line with these efforts, this study sought to examine how the purchase intention for low-sodium/low-sugar products vary based on consumers’ previous choices of low-sodium/low-sugar products and other consumer-related factors. For this study, two online survey-based experiments were conducted: one using soy sauce to represent a sodium-based product and the other using yogurt to represent a sugar-based product. The significant variables that influenced the purchase intention for both were the consumers’ previous low-sodium/low-sugar product choices and their propensity for food neophobia. Consumers who had previously selected regular products showed a lower intention to purchase low-sodium soy sauce or low-sugar yogurt. In addition, those who had a strong tendency toward food neophobia also had a significantly lower purchase intention for these products. Moreover, the lower the consumer′s unhealthy = tasty intuition (UTI), the higher the purchase intention for the low-sodium soy sauce, but UTI did not act as a significant variable for the low-sugar yogurt. These results demonstrate that government interventions for low-sodium products and low-sugar products should be differentiated.

## 1. Introduction

Sodium is an essential nutrient for the maintenance of human health. The recommended minimum daily intake of sodium is 500 mg [1]. However, excessive sodium intake has been identified as a health risk as it can lead to health problems such as stroke and cardiovascular disease [2]. The issue of excessive sodium intake is particularly problematic in South Korea since it has one of the highest sodium consumption rates in the world [1].

Similarly, while sugar has now become a major component of our daily nutrition [3], the excessive consumption of sugar is associated with the risk of weight gain and cardiometabolic problems [4]. And excessive sugar intake, as well as sodium intake is also a health problem in South Korea as the sugar-sweetened beverage (SSB) consumption rate among adults increased from 66% to 69% between 2001 and 2009 [5].

In response to these situations, the food industry has been reducing the sodium and sugar contents of products to meet government health guidelines [6]. However, food reformulation efforts alone are insufficient for inducing healthy consumption patterns among consumers. Depending on external and consumer-related factors, the results of these efforts can vary. Previous studies have shown that variables such as age, gender, income, health (whether they smoke, whether they have certain diseases, etc.), special diet, and price sensitivity have a significant impact on consumer attitudes toward low-sodium or-low-sugar products [7,8,9,10,11,12].

Therefore, this study investigates how consumers’ purchase intention for low-sodium/low-sugar products differs depending on their previous choices, level of unhealthy = tasty intuition (UTI), and food neophobia. As stimuli, we use soy sauce products to represent a sodium-based food category and yogurt products to represent a sugar-based food category. In comparison to regular products, we assume that the low-sodium and low-sugar versions of both the soy sauce and yogurt are relatively new to consumers, while the regular versions are familiar to consumers in South Korea.

## 2. Theoretical Framework

### 2.1. Cognitive Dissonance Theory

According to Festinger’s dissonance theory, two cognitions that are related to each other can be either consonant or dissonant. If the two cognitions are consonant, then one follows consistently with the other. However, if the two cognitions are dissonant, the opposite of one cognition is consistent with the other. When dissonance occurs, an individual experiences psychological discomfort, and in order to reduce this discomfort, the individual is motivated to avoid any information that is likely to aggravate this dissonance [13]. Some of the important research generated by cognitive dissonance theory can be classified as based on the free-choice paradigm, belief-disconfirmation paradigm, effort-justification paradigm, and induced-compliance paradigm [14].

Among these paradigms, we will focus on the free-choice paradigm and belief-disconfirmation paradigm in this study. According to the free-choice paradigm, the choice between similarly valued alternatives triggers a psychological tension mediated by the preferred aspects of the rejected alternative and the unpreferred aspects of the selected alternative [13]. This tension is relieved by reassessing the options after the choice has been made [15,16]. The concept that choice shapes preference has been widely accepted by many researchers for many years [17].

This idea was demonstrated in 1956 by an experiment conducted by psychologist Jack Brehm, and the results have been replicated numerous times since [18,19]. In Brehm’s experiment, participants were required to select one out of two similarly valued items and then evaluate the selected item. As a result, they rated the selected item as better than they did initially, while they rated the rejected item as worse [20]. 

According to the belief-disconfirmation paradigm, dissonance occurs when people are exposed to information that does not match their beliefs. If the dissonance is not diminished by altering one’s own beliefs, then the dissonance can lead to the rejection or refutation of the new information [14].

Following dissonance theory, the prior choice of a particular soy sauce or yogurt product will lead to a preference for this previously selected product. Therefore, even if new information is provided later regarding a low-sodium or low-sugar alternative, those who have already chosen a regular product will disregard this new information as it is dissonant to their existing preference. By referring to previous research that has stated that preference is almost identical to purchase intention and is a good predictor of it [21], it can be predicted that:

- H1a: Consumers who once chose a regular soy sauce will have a lower purchase intention for a low-sodium soy sauce regardless of additional information about the low-sodium soy sauce product.
- H1b: Consumers who once chose a regular yogurt will have a lower purchase intention for a low-sugar yogurt regardless of additional information about the low-sugar yogurt product.

### 2.2. Unhealthy = Tasty Intuition (UTI)

Many researchers have posited and identified the positive effects of providing consumers with easily identified healthy foods [22,23,24]. However, not all consumers will necessarily be more likely to buy certain products because they seem healthier [25]. For example, some consumers will not choose healthier products over less healthy ones within the same food categories. For those consumers, a food with relatively healthy names, such as salad are perceived as less tasty than the same food with relatively unhealthy names, such as pasta [26]. 

Connected to this rationale is the UTI that some people hold [27]. According to Raghunathan et al., UTI is a belief held by consumers that the healthiness and tastiness of a food product have a negative correlation. In other words, when information is provided to consumers indicating that a product is healthy, the worse they infer its taste. There are two main sources for this belief. First, this intuition is generated internally from a more general belief that there is an inverse relationship between utilitarian and hedonic values. Along with this internal source of UTI, mass media and personal communications instill consumers with views that are compatible with this intuition.

However, the relationship between UTI and its consequences may be more complicated than we think [26]. Furthermore, it is also important to highlight that the degree of UTI varies among consumers. Previous studies have indicated that there are consumers who are less likely to believe that unhealthy foods are tastier [26,28]. For instance, health-conscious consumers are less likely to base food decisions on UTI because they may place greater importance on health over taste [29,30]. To illustrate this, the taste expectations of dieters for healthy foods are more positive than those of non-dieters [26]. In fact, one previous study found an intuition opposite to UTI that exists in France in which healthy foods are believed to be tastier than unhealthy foods [31].

In the present study, UTI is indicated by the consumer tendency to evaluate healthy food as less tasty when given product information (nutrient contents, labels on the package, etc.). In this study, we consider healthy foods as those that are low in sodium or sugar. Therefore, it can be assumed that consumers with a lower UTI will have a higher purchase intention for low-sodium/low-sugar products. Thus, it is predicted that:

- H2a: Consumers with a lower UTI will have a higher purchase intention for a low-sodium soy sauce product.
- H2b: Consumers with a lower UTI will have a higher purchase intention for a low-sugar yogurt product

### 2.3. Food Neophobia

Underlying the relationship between a change in the taste of a product and the consumer acceptance of it is the feeling that consumers have when they are unfamiliar with the changed taste. In this context, the concept of food neophobia can be described as a consumer reluctance or unwillingness to try an unfamiliar product [32]. People expect novel foods to be unfavorable or even dangerous as compared to familiar ones, and this tendency lowers their willingness to try novel foods [33]. 

One of the ways to reduce food neophobia is to expose individuals to and increase familiarity with unfamiliar foods [34,35,36]. However, since the degree to which people are exposed to novel foods varies, attempts to reduce food neophobia by increasing familiarity or exposure should be made with caution [37]. Previous studies have revealed that there is a positive relationship between age and food neophobia [32,38,39]. Other variables, such as culture, education, and income, can also influence the degree of individual food neophobia [38,40,41,42,43]

In addition, the tendency for food neophobia can vary from person to person [32,44]. While food neophobics tend to reject and avoid unfamiliar foods without even trying them [45], food neophilics enjoy trying unknown foods [46,47].

In the food industry, it is sometimes inevitable that alterations in flavor will result from changes in the sodium/sugar content of a food product, and consumers must make a hedonic adjustment to the change in flavor in order to become accustomed to the reformulated (low-sodium/low-sugar) product [48]. As a result, this could influence the long-term consumer acceptance of a product [49]. 

Marketing strategies related to increasing consumer familiarity with novel foods have shown the potential to introduce these products into the market successfully [50]. Therefore, in order to ensure the market success of reformulated food products, such as with sodium or sugar-reduced foods, it is imperative to verify the purchase intentions of consumers for these products in consideration of their level of food neophobia. Therefore, we suggest the following hypotheses:

- H3a: Consumers with a lower tendency toward food neophobia will have a higher purchase intention for a low-sodium soy sauce product.
- H3b: Consumers with a lower tendency toward food neophobia will have a higher purchase intention for a low-sugar yogurt product.

## 3. Materials and Methods

### 3.1. Stimuli Materials

The two products chosen for the study were (1) soy sauce as a sodium-based product and (2) yogurt as a sugar-based product (Appendix A). The experiments were designed to reflect all the brands of products sold in the actual markets to increase external validity. To do so, pictures of the real products, as well as their information (brand, price, and volume) were given to the participants at the early stage of the experiment. Soy sauce was selected as a stimulus in the first experiment and used as a representative product for the sodium-based product category because it has traditionally been used in South Korea and is assumed to be one of the main reasons why Korea’s sodium consumption is one of the highest in the world [1]. Although soy sauce is recognized worldwide for its many health benefits as a fermented food, it also has the potential to cause high blood pressure and diabetes since it accounts for 20.8% of the daily sodium consumption in Korea [51]. In the case of sugar intake, yogurt is selected as a stimulus for this study because it is one of Korea’s major sugar sources, according to the Korea Ministry of Food and Drug Safety [52]. Therefore, yogurt will be presented to experiment participants as a sugar-based product in the second experiment. 

In order to select the soy sauce products for this study, brands with the highest sales were selected based on a report from the Korea Agro-Fisheries and Food Trade Corporation [53]. For the low-sugar yogurt products, on the other hand, the products with the highest sales volume on the E-Mart Mall, one of the largest Korean retail chains, were selected. 

### 3.2. Participants

The effects of consumers’ previous choices, UTI, and food neophobia on the purchase intention for a low-sodium soy sauce product and a low-sugar yogurt product were studied among Korean housewives. Housewives were selected as the subject of our study because they are the main decision-makers for soy sauce and yogurt purchases in a market shopping context. 

The data was collected from an online panel established by a leading online research company in the winter of 2018. Ages of the members of the panel ranged from their 20s to 50s and were all legally capable of consenting to participation. Participants of this study were at first gathered by answering the e-mail whether to participate in the study.

In the soy sauce experiment, respondents were asked whether they had bought soy sauce in the prior 6 months in order to screen out respondents who do not have recent experience of purchasing soy sauce products. Of the 2989 respondents, 1913 answered that they have experiences of buying soy sauce products in the prior 6 months. Then, 1913 respondents, who have bought soy sauce in the prior 6 months, were asked to choose one of the choice-set of regular soy sauce and low-sodium soy sauce products. Of the 1913 respondents, 1628 (85.10%) had chosen a regular soy sauce product and 285 (14.90%) had chosen a low-sodium soy sauce product. For the convenience and balance of the demographic composition of the two sample groups, however, 233 respondents out of 285 who had selected the low-sodium product and 233 out of 1628 respondents who had selected the regular product (total 466) were sampled and studied. The number of samples for each group was assigned to distribute the demographic data, such as age, evenly. The average age of the 466 respondents was 39.4 years old (SD = 10.8).

Likewise, for the yogurt experiment, respondents were also asked whether they had bought yogurt in the prior 6 months. 1070 out of 1401 answered that they have experiences of buying yogurt products in the prior 6 months. Of the 1070 respondents, 662 (61.87%) had chosen a regular yogurt product and 408 (38.13%) had chosen a low-sugar yogurt product. Among these, 239 out of 662 respondents who had selected a low-sugar yogurt product and 246 out of 1070 respondents who had selected a regular yogurt product (total 485) were sampled and studied. The number of samples for each group was assigned for the same reason as in the first experiment. The average age of the participants was 39.4 years old (SD = 10.6). 

### 3.3. Procedure

Both experiments were performed by an online survey. Prior to the survey, all of the respondents were told that the data and opinions gathered through the survey were confidential and to be used for research purposes only, following Section 33 of the Korean statistical law. Only participants who gave consent to participate in the survey pressed the “Next” button. Gathered data of the participants were non-identifiable and stored in the company’s database for 1 year.

The survey of the soy sauce experiment was performed based on the scenario that the respondent had to buy soy sauce at the market for preparing meals. The 466 respondents were provided with images of eight soy sauce products currently sold at the market, with each including brief information on its volume, price, and unit price. Among the eight products, two were low-sodium and six were regular products, and respondents were asked to choose one.

A similar design and procedure were applied in the yogurt experiment. The survey was also carried out based on the scenario that the respondent had to buy yogurt at the market. The 485 respondents were provided with images of seven yogurt products currently sold at the market, with each including brief information on its volume, price, and unit price. Among the seven products, three were low-sugar and four were regular products, and respondents were asked to choose one.

All of the respondents from both experiments were asked about their intention to purchase a low-sodium/low-sugar product. The study aimed to see how the sodium/sugar content of the product affects the consumer’s purchase intention of the product by providing them with additional information about low-sodium/sugar products immediately after the first choice of the participants. Those who had selected a low-sodium/low-sugar product in each experiment were given the information that the product they chose was low in sodium/sugar and were asked to evaluate their intention to purchase the product. On the other hand, those who had selected a regular product in each experiment were shown pictures and information for low-sodium/low-sugar products and were asked to evaluate their intention to purchase these products. The quality of the information given to both respondents in each experiment was controlled to be the same.

Next, the respondents from both experiments completed questionnaires regarding their UTI and food neophobia. Finally, basic demographic and personal information about the participants were taken in order to control the main independent variables. Information was collected about their interest in diet, family size, whether they have a family member suffering from atopy, high blood pressure, diabetes, or cancer, income, how often they cook at home, education level, and age.

### 3.4. Measures

Purchase intention was the main dependent variable in the study. To measure the purchase intention of a low-sodium/low-sugar product for each experiment, three items were rated on a 5-point scale (1 = ”very low” and 5 = ”very high”; Table 1) [54]. 

There were three main independent variables in this study: previous choice, UTI, and food neophobia. First, in order to measure the respondents’ previous choice, we divided the respondents into two groups based on whether they had chosen a low-sodium/low-sugar product or not. According to their choice, we assigned a dummy variable (PC) (Table 8). 

Next, to measure the participants’ intuitive belief that the less healthy the food, the better its inferred taste, that is the UTI, they were asked to rate three items [30] on a 5-point scale (1 = “strongly disagree” and 5 = ”strongly agree”; Table 2).

Finally, the participants answered 10 items for a food neophobia scale (FNS) [32] to measure their reluctance to eat and/or avoidance of novel foods based on a 5-point scale (1 = “strongly disagree” and 5 = ”strongly agree”; Table 3). The higher the FNS score, the weaker the consumer’s food neophobia, and the lower the FNS score, the stronger the food neophobia. All items were written in Korean.

### 3.5. Data Analysis

Before conducting a multiple regression analysis to analyze the effects of the independent variables on the consumers’ purchase intention for the products of the study, the validity of each individual item from the questionnaire was assessed. To test the validity of our constructs, we used structural equation modeling to perform a confirmatory factor analysis with SmartPLS 2.0 and a bootstrap resample procedure [55].

Reliability was confirmed with a Cronbach’s alpha. Generally, values that are greater than 0.70 represent a good measure [56]. Then, individual item loadings were used to check convergent validity. A loading of 0.70 or higher from a partial least square method is considered adequate. The validity of the measurement model was evaluated by the composite reliability (CR) index, partial least squares (PLS) factor loadings, and average variance extracted (AVE). A value greater than 0.70 CR means that a construct maintains both its internal consistency and convergent validity [57]. The recommended levels for PLS factor loadings and AVE are 0.70 and 0.50, respectively.

As can be seen in Table 4 and Table 5, the constructs from both experiments passed the reliability test since all of the Cronbach’s alpha values were higher than 0.70, indicating that the constructs have adequate internal consistency [56]. In addition, values greater than 0.70 for CR show that the constructs have both internal consistency and convergent validity [57].

This study used the square root of AVE (Table 6 and Table 7) and a cross-loading matrix to verify the discriminant validity of the measurement model. The square root should be larger than the correlations between the constructs [58]. The amount of variance shared between the latent variables indicates the presence of a strong discriminant validity [59]. Table 6 and Table 7 show that for both experiments, each construct has a higher correlation with the measures than the other.

Subsequently, a multiple regression analysis was conducted with the three main independent variables: whether the respondents had previously selected a low-sodium soy sauce or not (previous choice), UTI, and food neophobia. Calculations were performed using RStudio version 3.4.4.

## 4. Results

The results from the soy sauce experiment and the yogurt experiment are as follows (Table 8). First, the results from the soy sauce experiment revealed that among 466 respondents, those consumers who had previously chosen a regular soy sauce over low-sodium versions tended to have a lower purchase intention for a low-sodium product. Meanwhile, consumers who had a weaker intuition that delicious foods are not necessarily unhealthy (lower UTI) tended to have a higher purchase intention for a low-sodium soy sauce product. In addition, consumers who were less fearful of trying new foods (low food neophobia) tended to have a higher purchase intention for a low-sodium soy sauce product. These results support H1a, H2a, and H3a.

There were three control variables found in the results from the first experiment significant to the purchase intention for a low-sodium soy sauce: age, income, and diet interest. Consumers in their 20s and 30s had a lower purchase intention for a low-sodium soy sauce product compared to those who were in their 50s. On the other hand, consumers with a higher household income and consumers who were more interested in their diet had a higher purchase intention for a low-sodium soy sauce product.

The results from the yogurt experiment show that among 485 respondents, those consumers who had previously chosen a regular yogurt product over low-sugar versions tended to have a lower purchase intention for a low-sugar yogurt product. In addition, consumers who were less afraid of trying novel foods (low food neophobia) had a higher purchase intention for a low-sugar yogurt product. However, UTI was not a significant independent variable for the purchase intention for a low-sugar yogurt product. Therefore, the results support H1b and H3b but do not support H2b.

In the yogurt experiment, there were four control variables significant to the purchase intention for a low-sugar yogurt: age, diet interest, grocery shopping frequency, and family size. Consumers in their 20s had a lower purchase intention for a low-sugar yogurt product compared to those who were in their 50s. Older consumers, consumers who were more interested in their diet, and consumers who did their grocery shopping more frequently had a higher purchase intention for a low-sugar yogurt product. In addition, the larger the size of a consumer’s family, the higher the purchase intention for a low-sugar product.

## 5. Discussion

### 5.1. Consumers’ Previous Choice and the Purchase Intention for a Low-Sodium/Low-Sugar Product

The results from our study demonstrate that the previous choice shapes the preference of consumers. In other words, whether consumers had chosen a low-sodium/low-sugar product in the past or not make a significant impact on their purchase intention for these products. From the findings, we can draw some important implications.

In this study, respondents were asked to choose a product first from a choice set and were then informed about the low-sodium/low-sugar products. They were then asked to evaluate their purchase intention for these products. It should be noted that the presented information did not play a significant role in increasing the purchase intention for the low-sodium/low-sugar products. This is in line with our conjecture that those who had chosen regular products would disregard the information as it was dissonant with their preferences [14].

The findings suggest that governmental agencies should divide consumers into segments and implement different strategies. We suggest dividing consumers into segments of (1) consumers who have never purchased sodium/sugar-based products, (2) consumers who buy low-sodium/low-sugar products, and (3) consumers who buy regular products.

First, for the (1) consumers who have never purchased sodium/sugar-based products but are prospective consumers, governmental agencies should consider strategies to raise awareness of these products. It is imperative to add low-sodium/low-sugar products to the consumer choice set. Next, there is a need to encourage (2) consumers who are already a customer of low-sodium/low-sugar products to repurchase them. In order to do that, it is suggested that governmental agencies introduce recipes and put labels on the products to make consumers more informed and increase their involvement.

Finally, strategies that instill dissonant cognitions to the behavior of consuming high-sodium/low-sugar products can be considered for (3) consumers who are already consuming regular products. Following an example used by Festinger (1957)**,** some consumers may experience dissonance and divert their purchasing behaviors to reduce dissonance when they are faced with campaigns and public advertisements promoting that excessive sodium and sugar intake is bad for their health.

### 5.2. Consumers’ Unhealthy = Tasty Intuition and the Purchase Intention of a Low-Sodium/Low-Sugar Product

Consumers’ UTI made a significant impact on their purchase intention for a low-sodium soy sauce product. However, contrary to our expectations, it did not significantly influence the purchase intention for a low-sugar yogurt product. There may be some explanations for this. Soy sauce has a relatively high switching cost as it has a longer consumption period per purchase as compared to yogurt. In addition, soy sauce is not a product that is consumed directly, but rather, it is seasoning that adds saltiness during the cooking process. Thus, this saltiness itself is a highlight of soy sauce. Therefore, consumers with a high UTI will tend to have a significantly lower intention to purchase a low-sodium soy sauce product because purchasing it entails high uncertainty. On the other hand, yogurt is not a product eaten for sweetness alone, and therefore, UTI may not make a significant impact on consumer purchase intention in this case.

In addition, consumer perceptions of tastiness and healthiness can differ depending on the food product category [60]. Food products can be divided into a tastiness-associated food category and a healthiness-associated food category [61], yet some products can be perceived as having both tasty and healthy dimensions. In line with this idea, it is assumed that yogurt, unlike soy sauce, can be perceived as neutral in terms of healthiness and tastiness for consumers in Korea.

We can also speculate that Korea’s market environment affected the experiment results. Our data shows that 14.90% of the respondents selected a low-sodium soy sauce product and 85.10% selected a regular product. Meanwhile, the yogurt choices were more evenly distributed with 38.13% of respondents having selected a low-sugar yogurt product and 61.87% having selected a regular product. This suggests that consumer preconceptions about the taste of low-sugar yogurt have already disappeared or that there are already good tasting low-sugar yogurt products on the market, while there still exist preconceptions regarding the taste of low-sodium soy sauce.

Therefore, in order to increase consumers’ purchase intention for low-sodium products, especially for the soy sauce category, it is necessary to change the behavior of consumers in the long term by utilizing media-based promotion strategies. For example, media promotion strategies using contents such as specific and interesting sensory experiments and blind tests can be efficacious for overcoming consumer preconceptions that using low-sodium soy sauce will negatively affect the taste of food.

### 5.3. Consumers’ Food Neophobia and the Purchase Intention for a Low-Sodium/Low-Sugar Product

This study assumes that when a food product with lower sodium or sugar content is introduced by a widely accepted and long-established food brand, the consumer perceives the new product as novel relative to the original. Based on this assumption, we hypothesized that the weaker the food neophobia tendency of consumers, the stronger their purchase intention for low-sodium and low-sugar products. The results confirmed our hypothesis (H1a and H1b confirmed). One of the implications of this study is the interpretation of the consumer the consumption of soy sauce and yogurt products from a different perspective than previous studies. The study assumes that regular soy sauce and yogurt products are familiar to consumers, but are recognized as unfamiliar to consumers when the sodium and sugar contents are lower, respectively.

Although yogurt is a widely-consumed product category worldwide, soy-based foods, including soy sauce, are only recently being introduced to the Western world [62]. For this reason, many prior studies have been conducted in consideration of the factors that affect Western consumers (i.e., food neophobia) in their consumption of soy products, which would be novel foods from their perspective [63,64,65,66,67,68,69]. However, soybeans are a traditional food source in East and Southeast Asia [70]. In particular, soy sauce is a product that has traditionally been used in many dishes in Korea. Therefore, it is a more reasonable explanation that, while soy sauce itself is not a new food for Korean consumers, low-sodium soy sauce reformulated from the original is considered a new product for Koreans.

There have been other studies that have considered consumers’ purchase intention of yogurt products and the effects of food neophobia [71]. The yogurt items considered in those studies, however, were mainly functional products, such as probiotic yogurts [72,73]. However, the novelty covered in this study was not a novelty from adding a new nutritional ingredient to an existing product but rather from lowering the sugar content of a product already familiar to consumers.

The relationship between food neophobia and purchase intention for the products of our study indicates that alleviating consumer food neophobia for low-sodium/low-sugar content products may result in more positive consumer responses. Since a number of prior studies have demonstrated that repeated exposure to novels foods and flavors tends to enhance preferences [34,35,36], increasing the amount of exposure to these products can be one way to reduce food neophobia and encourage consumers to acquire preferences for low-sodium/low-sugar products. Therefore, giving out food samples or, as in the case of low-sodium soy sauce, making and distributing recipe books using the product can be suggested.

### 5.4. Control Variables

Control Variables that had significant impacts on the purchase intention of low-sodium soy sauce products were age, income, and diet interest. The result is in line with previous studies. According to the previous research [7], sodium content is considered significantly important in food purchases to older and middle-high income consumers. In addition, when it comes to the perception of low-sodium products, age, income, and family concern about diet-related problems are significant factors. Aged consumers with higher income levels and diet-related concerns within the family are more likely to recognize low-sodium products [8].

On the other hand, control variables that had significant impacts on the purchase intention of low-sugar yogurt products were age, diet interest, grocery shopping frequency, and family size. While consumers in their 20s and 30s had significantly lower purchase intention for low-sodium soy sauce products compared to those in their 40s and 50s, only consumers in their 20s had significantly lower purchase intention for low-sugar yogurt product. On the other hand, consumers with bigger family size had significantly higher purchase intention for low-sugar yogurt product while they were not for a low-sodium soy sauce product. According to the report from the Korea Agro-Fisheries & Food Trade Corporation report, the ages of the yogurt product consumers are evenly distributed compared to other dairy products [74]. As the number of family members increases, the age range of family members will also vary. In the case of a yogurt product, since the products are consumed by various ages compared to a soy sauce product, the increase in the number of family members would mean the increased demand for yogurt per household. This may explain why the Family size was a significant variable in the purchase intention of a low-sugar yogurt product compared to a low-sodium soy sauce product. In addition, according to a previous study, individuals who were more interested in losing their weights consumed low-sugar carbonated drinks such as Coca-Cola Light and Fanta Light significantly more than those who were not [75]. It is consistent with our finding that as consumers are more interested in their diet, their purchase intention for a low-sugar yogurt product is significantly higher. Furthermore, previous researches show that consumers who do grocery shopping more often tend to buy healthier foods [76], and college students who do not do grocery shopping tend to have poor eating habits, such as drinking more carbonated drinks or eating high-calorie food products compared to those who do grocery shopping at a convenience store or supermarkets [77]. These findings explain why grocery shopping frequency had a significant impact on the purchase intention of low-sugar yogurt products in this study.

### 5.5. Limitations and Further Research

Many different factors influence consumers’ purchase intention for low-sodium/low-sugar products, and it is impossible to consider all of these factors in a single study. This study focused on the influence of external factors, specifically previous choice experience, and consumer-related factors (UTI, food neophobia) on the purchase intention for these products. Other factors, such as product-related factors, need further research.

In addition, many different low-sodium food products exist in the sodium-based food product category, and there are also many low-sugar food products on the market. As stimuli, the present study used soy sauce to represent the sodium-based food product category and yogurt to represent the sugar-based food product category. More food product categories should be considered in order to generalize our findings in future research.

Furthermore, sodium-based and sugar-based food products differ not only in their sensory characteristics but also in the situations where they are consumed and the people who consume them. Further research is needed to determine the factors that influence the purchase intention of low-sodium and low-sugar food products based on the qualitative differences between these two product categories. For instance, comparing sodium-based products and sugar-based products that are perceived in terms of the same dimension (healthiness vs. healthiness, tastiness vs. tastiness) can be one way to consider the qualitative differences of these two food product categories.

Moreover, in conducting online purchasing experiments and surveys with low-sodium soy sauce and low-sugar yogurt products and analyzing the results, it was difficult to control for variables such as brand, price, and volume due to the structure of the survey. There was a trade-off between internal validity on the one hand and external validity on the other hand in the experimental design process. In future experiments, there is a need to control the impact of these variables on consumer choice and purchase intention while conducting the surveys and analyzing the results.

Finally, this study was conducted mainly on the group that can be surveyed online easily due to the nature of the online survey. Therefore, there is a possibility that the research results could be age-biased because the panel members who participated in the survey were limited to their 20s to 50s. Future research needs to focus on recruiting samples to represent all ages.

## 6. Conclusions

This study has its originality and value in that the study has interpreted the consumer consumption of soy sauce and yogurt products from a different perspective than previous studies. The study begins with the assumption that regular soy sauce and yogurt products are familiar to consumers, but are recognized as unfamiliar to consumers when the sodium and sugar content is reformulated, respectively. This way, the present work helps expand the view of defining a new food product.

This study aimed to examine the consumer’s previous choice of low-sodium/sugar products, unhealthy = tasty intuition and the propensity of food neophobia influence their intention to purchase low-sodium/sugar products. The result turned out that the previous choice of regular soy sauce and yogurt products had a negative effect on the purchase intention for low-sodium soy sauce and low-sugar yogurt products. In addition, consumer’s strong UTI had a negative influence on the purchase intention for low-sodium soy sauce products, but no significant influence on that of low-sugar yogurt products. Furthermore, consumer’s stronger propensity for food neophobia had a negative effect on the purchase intention for both low-sodium soy sauce products and low-sugar products. The result also suggests that not only consumer’s previous experience and their disposition, but also demographic factors such as age, income, diet interest, and family size have an impact on purchase intention for these low-sodium/sugar products. Furthermore, it can be said that the factors have different impacts on the purchase intention depending on the product category and whether sodium or sugar is the main content of the product.

## Figures and Tables

**Table 1:** Items used in the purchase intention (PI) scale.

PI	Description
PI 1	I am considering purchasing the product.
PI 2	I will purchase the product as soon as possible.
PI 3	If there is a chance, I have the intention to buy the product.

**Table 2:** Items used in the unhealthy = tasty intuition (UTI) scale.

UTI	Description
UTI 1	Things that are good for me rarely taste good.
UTI 2	There is no way to make food healthier without sacrificing taste.
UTI 3	Healthy food is usually less tasty.

**Table 3:** Items used in the food neophobia scale (FNS).

FNS	Description
FNS 1	I am constantly sampling new and different foods.
FNS 2	I don’t trust new foods. (R)
FNS 3	If I don’t know what is in a food, I won’t try it. (R)
FNS 4	I like foods from different countries.
FNS 5	Ethnic foods look too weird to eat. (R)
FNS 6	At different parties, I will try new foods.
FNS 7	I am afraid to eat things I have never had before. (R)
FNS 8	I am very particular about the foods I will eat. (R)
FNS 9	I will eat almost anything.
FNS 10	I like to try new ethnic restaurants.

Items for which scoring is reversed are marked (R).

**Table 4:** Reliability and validity values from the first experiment: soy sauce products.

Constructs	Item	Factor Loading	Average Variance Extracted	Composite Reliability	Cronbach’s Alpha
Unhealthy = tasty intuition (UTI)	UTI 1	0.9021	0.8339	0.9377	0.9013
	UTI 2	0.9298			
	UTI 3	0.9074			
Food neophobia (FNS)	FNS 2	0.8295	0.7184	0.8841	0.7184
	FNS 4	0.9131			
	FNS 5	0.7959			
Purchase intention for a low-sodium soy sauce product (PI)	PI 1	0.8542	0.7504	0.9000	0.7504
	PI 2	0.8216			
	PI 3	0.9201			

**Table 5:** Reliability and validity values from the second experiment: yogurt products.

Constructs	Item	Factor Loading	Average Variance Extracted	Composite Reliability	Cronbach’s Alpha
Unhealthy = tasty intuition (UTI)	UTI 1	0.9216	0.7784	0.9129	0.8773
	UTI 2	0.7956			
	UTI 3	0.9234			
Food neophobia (FNS)	FNS 2	0.7278	0.6126	0.8629	0.7882
	FNS 4	0.8751			
	FNS 5	0.7579			
	FNS 8	0.7621			
Purchase intention for a low-sodium soy sauce product (PI)	PI 1	0.9055	0.7869	0.9171	0.8656
	PI 2	0.9018			
	PI 3	0.8528			

**Table 6:** Correlations of the latent variables and the square root of the average variance extracted from the first experiment: soy sauce products.

	(1)	(2)	(3)
(1) Unhealthy = tasty intuition	(0.9132)		
(2) Food neophobia	−0.1857	(0.8476)	
(3) Purchase intention for a low-sodium soy sauce product	−0.1482	0.0983	(0.8663)

**Table 7:** Correlations of the latent variables and the square root of the average variance extracted from the second experiment: yogurt products.

	(1)	(2)	(3)
(1) Unhealthy = tasty intuition	(0.8823)		
(2) Food neophobia	−0.1126	(0.7827)	
(3) Purchase intention for a low-sodium soy sauce product	−0.0503	0.1723	(0.8871)

**Table 8:** Multiple regression analysis results.

		Purchase Intention for a Low-Sodium Soy Sauce Product		Purchase Intention for a Low-Sugar Yogurt Product	
Type	Independent Variable	Stan.B	*p*-Value	Stan.B	*p*-Value
Previous Choice	PC	0.2905	0.0000 ***	0.3554	0.0000 ***
Unhealthy = tasty intuition	UTI	−0.1067	0.0163 *	0.0007	0.9875
Food neophobia	FNS	0.0955	0.0334 *	0.1214	0.0048 **
Control Variable	Age_20 ^a)^	−0.2106	0.0003 ***	−0.1730	0.0011 **
	Age_30 ^a)^	−0.1409	0.0119 *	−0.0580	0.2756
	Age_40 ^a)^	−0.0665	0.2195	−0.0895	0.0826
	Income	0.0863	0.0780	−0.0416	0.3644
	Diet Interest	0.1440	0.0011 **	0.1019	0.0166 *
	Home Cooking interest	0.0365	0.4376	0.0593	0.1757
	Education	−0.0453	0.3293	0.0225	0.5960
	Grocery shopping frequency	0.0492	0.2580	0.1372	0.0011 **
	Family size	0.0098	0.8434	0.1364	0.0039 **
	**R^2^**	0.1530		0.1851	
	**F-statistic**	8.0000		10.1600	
	***p*** **-value**	0.0000		0.0000	

*** *p* < 0.001, ** *p* < 0.01, * *p* < 0.05; ^a)^ Based on a dummy variable that was coded as 1 if the respondent belongs to this age range. All control variables except for the age variables are continuous variables.

**Figure A1:**
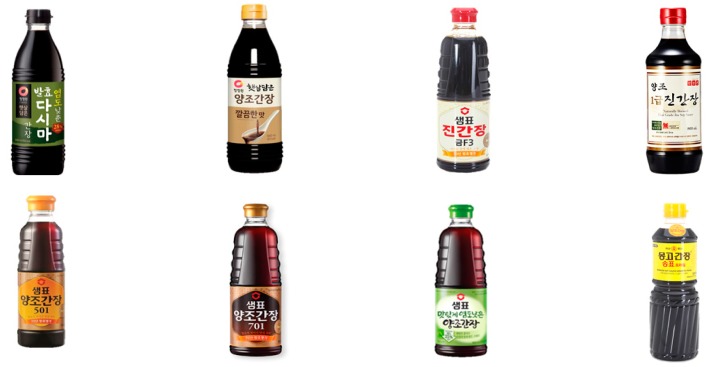
Soy sauce products used as stimuli in the survey.

**Figure A2:**
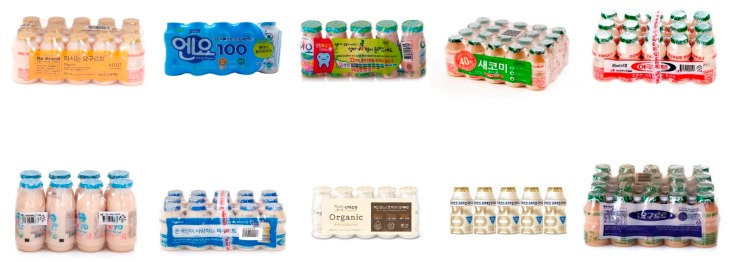
Yogurt products used as stimuli in the survey.
